# Transcriptome response of roots to salt stress in a salinity-tolerant bread wheat cultivar

**DOI:** 10.1371/journal.pone.0213305

**Published:** 2019-03-15

**Authors:** Nazanin Amirbakhtiar, Ahmad Ismaili, Mohammad Reza Ghaffari, Farhad Nazarian Firouzabadi, Zahra-Sadat Shobbar

**Affiliations:** 1 Department of Agronomy and Plant Breeding, Faculty of Agriculture, Lorestan University, Khorramabad, Iran; 2 Department of Systems Biology, Agricultural Biotechnology Research Institute of Iran (ABRII), Agricultural Research, Education and Extension Organization (AREEO), Karaj, Iran; Jawaharlal Nehru University, INDIA

## Abstract

Salt stress is one of the major adverse environmental factors limiting crop productivity. Considering Iran as one of the bread wheat origins, we sequenced root transcriptome of an Iranian salt tolerant cultivar, Arg, under salt stress to extend our knowledge of the molecular basis of salinity tolerance in *Triticum aestivum*. RNA sequencing resulted in more than 113 million reads and about 104013 genes were obtained, among which 26171 novel transcripts were identified. A comparison of abundances showed that 5128 genes were differentially expressed due to salt stress. The differentially expressed genes (DEGs) were annotated with Gene Ontology terms, and the key pathways were identified using Kyoto Encyclopedia of Gene and Genomes (KEGG) pathway mapping. The DEGs could be classified into 227 KEGG pathways among which transporters, phenylpropanoid biosynthesis, transcription factors, glycosyltransferases, glutathione metabolism and plant hormone signal transduction represented the most significant pathways. Furthermore, the expression pattern of nine genes involved in salt stress response was compared between the salt tolerant (Arg) and susceptible (Moghan3) cultivars. A panel of novel genes and transcripts is found in this research to be differentially expressed under salinity in Arg cultivar and a model is proposed for salt stress response in this salt tolerant cultivar of wheat employing the DEGs. The achieved results can be beneficial for better understanding and improvement of salt tolerance in wheat.

## Introduction

Soil salinity is a major environmental factor which limits the growth and development of plants, resulting in decrease in crop productivity and quality[1, 2]. It is estimated that salt stress affects approximately 20% of the irrigated land worldwide and will lead to the loss of 50% of cultivable land by the middle of the twenty-first century[3].

High soil salt concentrations reduce the capability of a plant to absorb water. Moreover, when Na^+^ and Cl^−^ are absorbed in large quantities by roots, both Na^+^ and Cl^−^ adversely influence growth by ruining metabolic processes and reducing photosynthetic efficiency[4, 5]. Therefore, salt stress limits the growth of plants through early-occurring osmotic stress and slowly-occurring ion cytotoxicity[6]. Plants use mechanisms to relieve osmotic stress by decreasing water loss and maximizing water uptake. In addition, plants minimize the adverse consequences of ionic Na^+^ stress by excretion of Na^+^ from leaf tissues and by compartmentalization of Na^+^ largely into vacuoles[7, 8]. Notwithstanding these tolerance mechanisms, salt stress declines crop yields and results in continuous loss of arable land. Therefore, identifying the main genes and mechanisms involved in salinity tolerance in order to engineer crops to improve salt-tolerance mechanisms is necessary to address these challenges[6].

Clarifying the key components in the plant salt tolerance network is essential to engineer more salt tolerant plants. Three kinds of genes are involved in salt stress response in plants including the genes involved in sensing and signaling of the stress, transcriptional regulators and salt-stress related genes. Under salt stress, Na^+^ enters the cell through non-selective cation channels and other membrane transporters (that most of them are unknown) and inside the cell, Na^+^ is sensed by an unknown sensory mechanism. At the next step, Ca^2+^, reactive oxygen species (ROS) and hormones act as the secondary messengers. In the Ca^2+^ signaling pathway, for example, kinases like Calcineurin B-like proteins (CBLs), CBL-interacting protein kinases (CIPKs) and Calcium-dependent protein kinases (CDPKs) are present which can change the transcriptional profile of the plant. Transcription factors families such as WRKY, MYB, bHLH, bZIP, AP2/ERF and NAC had been shown to be engaged in salt stress response. Finally, these early signaling pathways lead to expression and activation of cellular detoxification mechanisms including Na^+^ transport mechanisms and osmotic protection strategies[6].

RNA-seq, having high accuracy and sensitivity is one of the most suitable techniques to study the whole transcriptome[9, 10]. It has advantages such as the ability to identify novel genes/transcripts, detect low abundance transcripts, identify genetic variants and detect more differentially expressed genes with higher fold-change in comparison with microarray[10, 11]. Recently, a few RNA-seq studies was used to explore the transcriptome of bread wheat under salt stress: Goyal et al. (2016) found genes involved in providing energy to form proton gradient to drive exceeding cytoplasmic Na^+^ into vacuoles (like V-ATPase gene), ROS scavengers, genes engaged in energy metabolism adjustment (like ATP citrate synthase) and signaling genes (like Cbl-interacting protein kinase) as the most important genes involved in salt tolerance in root transcriptome of Kharchia local variety[12]. Zhang et al. (2016) introduced some genes such as a NAC transcription factor (homologous to Arabidopsis AtNAC025), a histone-lysine N-methyltransferase (homologous to Arabidopsis AtSDG16), a MYB transcription factor (homologous to Arabidopsis AtMYB333) and *TaRSL4* gene (positively associated with root hair development) as necessary genes for salt stress tolerance in bread wheat root[13]. Xiong et al. (2017) compared salt responsive transcriptome of shoot between a salt tolerant bread wheat mutant and the salt sensitive wild type and showed that homeostasis of oxidation-reduction process is important for salt tolerance. They also found “Butanoate metabolism” as a new pathway for salinity response. Moreover, they found key genes for salinity tolerance such as arginine decarboxylase, polyamine oxidase and hormones-related genes to be more induced in salt-tolerant genotype[14]. In spite of the precious insight into the molecular and cellular mechanisms by which bread wheat responds to and tolerate salinity found by these recent research studies, the regulatory mechanisms engaged in harmonizing salt stress tolerance and plant growth are not completely perceived. Thus, a better understanding of salt-tolerance mechanisms would be helpful for breeding salt-tolerant wheat cultivars, in order to stabilize wheat production.

Wheat is the most important crop in Iran and the staple food for most of the people. Given that Iran is known as one of the origins of bread wheat and its wild progenitors [15–18], sequencing an Iranian salt tolerant wheat cultivar (such as Arg) can provide new and valuable information. Therefore, in this study, we used the Illumina sequencing to compare the transcriptome of Arg under normal and salt-stressed conditions.

In order to analyze the bread wheat RNASeq data, TGACv1 reference genome of *T*. *aestivum* was used. To date, this is the most complete and accurate sequence assembly and annotation of the bread wheat reference accession, Chinese Spring[19]. To the best of our knowledge, it is the first time that this reference genome is employed for RNASeq data analysis under salt stress. The differential gene expression patterns and alternative splicing events were analyzed. In addition, novel transcripts in response to salt stress were identified. Functional categorization of the DEGs was also carried out to determine different metabolic pathways engaged in salt stress response. Overall, this research provides a comprehensive overview of transcriptional regulation in bread wheat under salt stress.

## Materials and methods

### Plant growth and salt stress treatment

Seeds of bread wheat cultivars of Arg (salt tolerant) and Moghan3 (salt sensitive) were collected from Seed and Plant Improvement Institute, Karaj, Iran. Arg has been produced by the hybridization between cymmit cultivar of Inia and the salt tolerant local line of 1-66-22 in the Seed and Plant Improvement Institute (SPII) of Iran [20].

The seeds were surface sterilized in 1% Sodium hypochlorite (NaClO) for 10 min, then washed in distilled water several times, and finally laid on moistened filter paper. After 2–3 days, the uniformly germinated seeds were grown hydroponically in half-strength Hoagland solution in the green house. The 3-week old plants were salt treated using 150 mM NaCl solution for 12 h. The root samples were taken from both control and salt-treated plants with four biological replicates, each containing three plants. The root samples were immediately frozen in liquid nitrogen and stored at -80°C until analysis.

### RNA extraction and Illumina deep sequencing

Total RNA was isolated from the four biological replicates of normal and stressed roots (after 12 h exposure to salt stress) using RNeasy Plant Mini Kit (Qiagen) according to the manufacturer’s instructions (Qiagen). Equal amounts of total RNA of each two biological replicates were pooled for the RNA sequencing. The purity and integrity of RNA was checked by nanodrop, agarose gel electrophoresis and Agilent Bioanalyzer 2100 system (Agilent Technologies Co. Ltd., Beijing, China). The samples with RIN value higher than 9.1 were used for sequencing.

After quality control (QC) of the RNA samples, poly(A) enrichment, RNA fragmentation, random hexamer-primed cDNA synthesis, linker ligation, size selection and PCR amplification were done to prepare cDNA libraries for each sample. Finally, the qualified libraries were fed into HiSeq sequencers after pooling according to its effective concentration and expected data volume. The libraries were sequenced at the Novogene Bioinformatic Institute (Beijing, China) on an Illumina Hiseq 2500 platform and 150bp paired end reads were generated. After sequencing, reads containing adapters, reads with N > 10% (N indicates that the base cannot be determined) and reads having low quality (Qscore< = 5) base, which was over 50% of the total base, were removed.

### Quality control and reference-based assembly

The quality of raw fastq data was checked using FastQC toolkit. The high quality reads were submitted for mapping analysis against the release-34 version of wheat reference genome (ftp://ftp.ensemblgenomes.org/pub/release-34/plants/fasta/triticum_aestivum/dna/) using Tophat with default parameters. Assembly was done through Cufflinks using the TopHat mapping files with default parameters. Cuffmerge with default options was used to merge the individual assemblies and produce the final assembly. Furthermore, the novel transcripts were identified by Cuffmerge[21]. The assembled sequences were aligned against the NCBI non-redundant protein database through BlastX with an e-value cut-off of 1e^-3^ using Blast2GO program.

### Identification of differentially expressed genes (DEGs)

The FPKM method was used to calculate the gene/transcript expression in this research. Differential gene expression was defined using Cuffdiff available in Cufflinks package utilizing options,–upper-quartile-norm,–total-hits-norm and–frag-bias-correct. The genes with log2 fold change ≥ 1 (up-regulated genes) and ≤(− 1) (down-regulated genes) with Q-value cut off of ≤ 0.01 were considered as significant differentially expressed transcripts.

### Functional annotation and pathway analysis of DEGs

Classification of DEGs to GO terms was done using Blast2GO program at p-values < 0.05 [22]. Online KEGG Automatic Annotation Server (KAAS), http://www.genome.jp/kegg/kaas [23], using single-directional best hit (SBH) method was used to assign KEGG pathways to the DEGs. Furthermore, Mapman (version 3.5.1; http://mapman.gabipd.org/web/guest) [24] was used for pathway analysis of DEGs with P-value cut-off of ≤ 0.05. The DEGs were mapped on Arabidopsis pathway genes to characterize the genes involved in specific pathways.

### Alternative splicing analysis

Determination of alternative splicing events was done using AStalavista web tool (version 3; http://genome.crg.es/astalavista/) with default parameters. The outputs prepared for all the AS events were further analyzed manually.

### Quantitative Real Time PCR (qRT-PCR) validation

Three replicates were used for quantitative Real-Time PCR. cDNA was synthesized using qScript cDNA Synthesis Kit (Quantabio, USA) according to the manufacturer’s instruction. qRT-PCR was performed for three biological replicates using a LightCycler 96 Real-Time PCR System (Roche Life Science, Germany) and SYBR Premix EX TaqII (Takara Bio Inb,Japan) according to the manufacturer’s instructions. Normalization was done using Actin as an internal control gene as reported in previous studies[12, 24].

The gene specific primers are listed in S1 Table. The relative expression levels of the selected genes were calculated from cycle threshold values using the 2^−ΔΔCt^ procedure [25].

## Results and discussion

### Sequencing statistics

To obtain a better understanding of the mechanism underlying salt tolerance of Arg cultivar at global transcriptional level, the transcriptome of Arg plants grown in control and NaCl supplemented media were surveyed by RNA-Seq. In total, more than 113.34 million reads were produced. After trimming adapters and filtering out low quality reads, more than 111.73 million clean reads remained for further analysis. Among all the reads, more than 91.3% had Phred-like quality scores at the Q30 level (an error probability of 0.1%) (S2 Table). These results indicated that the quality of sequencing data is high enough for subsequent analysis. The transcriptome raw data of the present research have been submitted at SRA (Sequence Read Achieve) of NCBI with the accession numbers of SRR7755529, SRR7755530, SRR7755531 and SRR7755532.

Reference-based transcriptome assembly was accomplished via TopHat-Cufflinks pipeline[21] utilizing the wheat genome sequence as reference. The high quality reads were mapped to the release-34 version of wheat reference genome (ftp://ftp.ensemblgenomes.org/pub/release-34/plants/fasta/triticum_aestivum/dna/) and the alignment results showed that 85.4–86.4% of the total reads mapped to the wheat reference genome including 74.73–81.03% are uniquely matched (Table 1). The assembly of mapped reads led to the identification of a total of 203080 transcript isoforms and 104013 genes.

**Table 1:** Summary of Illumina transcriptome reads mapped to the reference genes (The numbers listed in the table are the sum of left and right reads).

Reads mapping	Reads number (%)			
	Control-rep1 Control-rep2 Salt-stressed-rep1 Salt-stressed-rep2			
Total reads	55051964	50888292	59933654	57599222
Total mapped reads	47295192(85.9%)	43513604(85.5%)	51183812(85.4%)	49765796(86.4%)
Unique match	44609343(81.03%)	40473327(79.53%)	47943836(79.99%)	43045674(74.73%)
Multi-position match	2685849(4.87%)	3040277(5.97%)	3239976(5.41%)	6720122(11.67%)
Total unmapped reads	7756772(14.1%)	7374688(14.5%)	8749842(14.6%)	7833426(13.6%)

### Identification of novel transcripts

One of the major advantages of RNA-seq analysis is to identify novel genes/transcript isoforms [9, 10, 26]. In this study, 26171 novel transcript isoforms and 15060 novel genes were identified. It was found that the average length of novel transcripts was lesser (1690 bp) than that of known (annotated) transcripts (2285bp) similar to what was reported in other plants such as rice and maize[27, 28]. Around 74% of the total assembled transcripts and more than 49.4% of the novel transcripts were assigned with a putative function (S3 Table).

GO analysis of novel transcripts revealed that in biological process category, the novel genes were involved in cellular process, biological regulation, localization, single organism process, metabolic process, cellular component organization or biogenesis, response to stimulus and regulation of biological process (S4 Table). In the molecular function category, most of the novel transcripts were involved in heterocyclic compound binding, small molecule binding, hydrolase activity, ion binding, transferase activity, carbohydrate derivative binding, organic cyclic compound binding, nucleotide binding, hydrolase activity, transferase activity and kinase activity (S5 Table). Furthermore, novel transcripts belonged to cytoplasm, cytoplasmic part, integral component of membrane, intracellular membrane-bounded organelle, plastid, nucleus and mitochondrion cellular component GOSlim categories (S6 Table).

### Identification of DEGs

**Fig 1:**
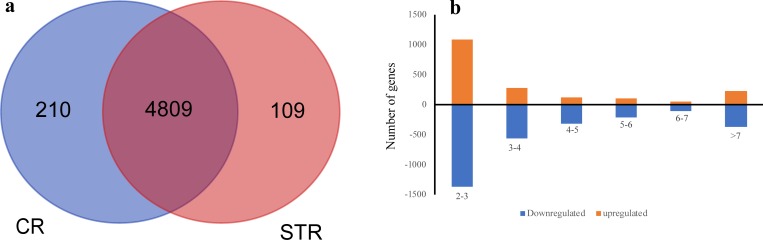
Survey of differentially expressed genes (DEGs) between control and salt stress in bread wheat. (Cut off pvalue: 0.01). (a) Out of 5128 DEGs, 109 genes were uniquely expressed in control and 210 genes were uniquely expressed under salt stress (b) Fold change distribution of 4809 DEGs available in both samples.

A total of 5128 genes were differentially expressed between salt treated and control bread wheat (Arg cultivar) root samples (S1 Fig). Among these DEGs, 1995 genes were up-regulated and 3133 genes were down-regulated under salt stress. In addition, 109 and 210 genes were unique in salt treated and control plants, respectively (Fig 1A). Among the genes exclusively expressed under salt stress, some important genes were observed which are known to be engaged in abiotic stress response such as transcription factors (e.g. AP2/ERF and MYB), LEA proteins, dehydrins, BURP domain-containing proteins and the genes involved in cell redox homeostasis (S7 Table). Assessing fold change distribution of the DEGs showed that most of the genes had a fold change between 2 and 3 and the least number had a fold change of 6–7 (Fig 1B).

As mentioned in the introduction, three kinds of genes are involved in salt stress response. The first group contains salt responsive genes, which are involved in sensing and signaling of the (S8 Table) were discovered among the up-regulated DEGs which may act as candidate osmosensors in *T*. *aestivum* under salt stress[29]. The molecular nature of Na^+^ sensors is yet unclear, the plasma membrane Na^+^/H^+^ antiporter Salt Overly Sensitive1 (SOS1) can be a possible candidate because its cytoplasmic end is assumed to be involved in Na^+^ sensing. The gene encoding SOS1 (S8 Table) was found among the up-regulated DEGs which may function as Na^+^sensor[30].

Fluctuation in the cytosolic calcium concentration is one of the primary responses to different stimuli, and elements involved in Ca^2+^ transport actively take part in retaining this flux and homeostasis[31]. Among the DEGs, there were 3 genes coding for calcium-transporter ATPases. One of them is the up-regulated novel gene which locates in TGACv1_scaffold_641741_U:17189–17824. Orthologue of the mentioned gene in rice is *Os*.*ACA7* (Os10g0418100), which locates in Golgi apparatus and is activated by Calmodulin[31]. Up-regulation of the Ca^2+^-ATPases in different plant species such as tomato, tobacco, soybean [32–35]and Arabidopsis has been reported under salt stress and they may help in dropping the cytosolic calcium level, that was elevated by NaCl stress, and the maintenance of Ca^2+^ homeostasis[31]. Also, overexpression of N-terminal modified ACA4 in Arabidopsis seedlings resulted in increased salt tolerance in comparison with wild-type plants[36]. Annexins constitute another group of Ca^2+^ transporters[31]. Annexin D4 is one of the Ca^+2^ transporters found in this study. Annexins operate downstream of the plasma membrane NADPH oxidases that produce extracellular hydroxyl radicals, which are able to activate Ca^+2^ influx through annexins [37]. Another Ca^2+^ transporter discovered in this study was a Na^+^/Ca^2+^ exchanger coded by a novel gene, located in GACv1_scaffold_571144_7AS:15506–18943 (*Ta*.*NCL2*). It has been reported that AtNCL localizes in the cell membrane, binds Ca^2+^ and takes part in Ca^2+^ homeostasis under abiotic stresses in Arabidopsis[38]. *Ta*.*GLR*, which encodes a Glutamate receptor, is another gene involved in Ca^2+^ transport in this study. Glutamate receptors are non-selective cation channels [31]and are responsive to abiotic stresses[39, 40].

Following increase in Ca^2+^concentration under salt stress, kinases like Calcium-dependent protein kinases (CDPKs)[41], calcineurin B-like proteins (CBLs) and CBL-interacting protein kinases (CIPKs)[42] may become activated, which are able to transduce the signal to downstream protein activity and gene transcription[6]. 19 DEGs encoding CIPKs were discovered (S8 Table), among which 6 genes had been demonstrated to be engaged in salt stress response based on the information available about their orthologues in Arabidopsis [43–45] (S9 Table). Six genes encoding Calmodulin were also found among the DEGs (S8 Table). One of the Ca^2+^-sensing proteins is Calmodulin (CaM) and it has been shown that CaM is involved in transduction of Ca^2+^signals. After interacting with Ca^2+^, CaM is subjected to conformational changes and affects the activities of CaM-binding proteins. A number of CaM-binding proteins were supposed to be involved in stress responses in plants, indicating the central role played by CaM in adaptation to detrimental environmental conditions[46].

Among the DEGs, many transcription factors (TFs) were discovered, proposing that TFs play important roles in salt stress response via regulating transcription of the downstream genes responsible for plant tolerance to salt stress. Transcription factors such as AP2/ERF, bZIP, Zn-finger, NAC, MYB and WRKY had been observed to be engaged in the regulation of abiotic stress tolerance in plants and a subset of these transcription factors are discussed in this study[47].

NAC genes are a class of plant-specific transcription factors containing a highly conserved N-terminal domain known as the NAC domain. In this study, 53 NAC domain containing genes were discovered among the DEGs, of which 29 NAC genes were up-regulated and 24 of them were down-regulated under salt stress (S8 Table). Some of these NAC genes have been defined to be involved in salt stress response based on the previous studies in wheat or on the basis of the information regarding their orthologues in Arabidopsis [48–51](S9 Table).

Another class of transcription factors are zinc finger proteins (ZFPs), among which four ZFP families of C2H2, CCCH, C3HC4 and C4 play many important regulatory roles in development, growth, stress response and phytohormone response in plants[52]. In this study, about 30 differentially expressed ZF transcription factors were identified which, based on their orthologues in the Arabidopsis, seven salt responsive members were predicted (S9 Table). Among these genes, AtSZF2 (orthologue of TRIAE_CS42_3AL_TGACv1_196305_AA0659290 in Arabidopsis) negatively regulate the expression of salt-responsive genes and play key roles in modulating salt stress tolerance in Arabidopsis plants.[26]

The MYB family, found in all eukaryotes, includes a large and functionally diverse classes of proteins. Most of the MYB proteins function as transcription factors and have been proved to be engaged in regulating different cellular processes, including biotic and abiotic stress response[53]. Based on the achieved results, 48 MYB transcription factors are induced by salt stress.

The third group of salt stress responsive genes are those involved in stress adaptation. Among the DEGs, there were genes coding for Aquaporins (controlling transport of water and ions)[54], LEA proteins, dehydrins and organic osmolytes such as proline in response to osmotic stress. Late-embryogenesis-abundant (LEA) proteins are induced by osmotic stresses in vegetative tissues and cause dehydration tolerance in vegetative tissues of plants[55]. Twenty seven genes coding for LEA proteins were discovered in the DEGs identified in this study. Production of LEA proteins alongside accumulation of organic osmolytes plays key roles in sustaining the low intracellular osmotic potential of plants and thereby attenuates the detrimental effects of salinity stress[56, 57]. Furthermore, peroxidases, catalases, glutaredoxins and Gluthatione-S- transferases were differentially expressed in response to oxidative stress caused by salinity.

For dealing with the ionic stress arising from salinity, the genes coding for plasma membrane Na^+^/H^+^ antiporter SOS1, K^+^ transporters and ABC transporters were available among the up-regulated DEGs. The ABC transporter, *Ta*.*ABAC15*, found among the up-regulated DEGs is involved in K^+^ uptake, and K^+^ / Na^+^ homostasis, based on the information about its Arabidopsis orthologue (At1g04120)[58].The genes coding for HAK potassium transporters were also discovered among the DEGs in this study. Horie et al. showed that overexpression of rice Na^+^-impermeable K^+^ transporter (*OsHAK5*) led to salinity tolerance in tobacco bright yellow 2 (BY2) cells[59]. Ion homeostasis during salinity stress needs the preservation of stable K^+^ attainment and distribution[60] given that K^+^ accumulation in plant cells equilibrates the poisonous effects of Na^+^ accumulation.

The gene coding for salt overly sensitive 1 (SOS1) was also discovered among the up-regulated DEGs in this research. Plasma membrane-localized SOS1 Na^+^/H^+^ antiporter[2], which exports Na^+^ out of the cell, besides tonoplast-localized NHX1 Na^+^/H^+^ antiporter[61] are the two main factors that sustain low cytoplasmic Na^+^ concentrations in plant cells[6]. As mentioned above, SOS1 besides functioning as antiporter to export Na^+^ out of the cell, may act as Na^+^ sensors, too. At the present study, SOS2 was not observed among the DEGs but the CIPK gene which its Arabidopsis orthologue is SOS2-like protein kinase PKS12 was available among the up-regulated DEGs which is engaged in salt stress response in Arabidopsis[44]. It is likely that this gene controls the activity of the Na^+^/H^+^ antiporter SOS1. Although the gene coding for Na^+^/H^+^ antiporter NHX1 was not available among the DEGs, but hexokinase1 which is able to phosphorylate NHX1 and increase its stability was discovered among the up-regulated DEGs. In fact, this gene increases salt tolerance through increasing compartmentalization of Na^+^ into vacuole[62].

### Gene ontology enrichment analysis for DEGs

In order to study the functions of DEGs, GO terms were extracted utilizing Blast2GO tool and then were exposed to GO enrichment analysis[22]. Annotation of DEGs revealed that a total of 4056(out of 5028) genes were assigned GO terms.

**Fig 2:**
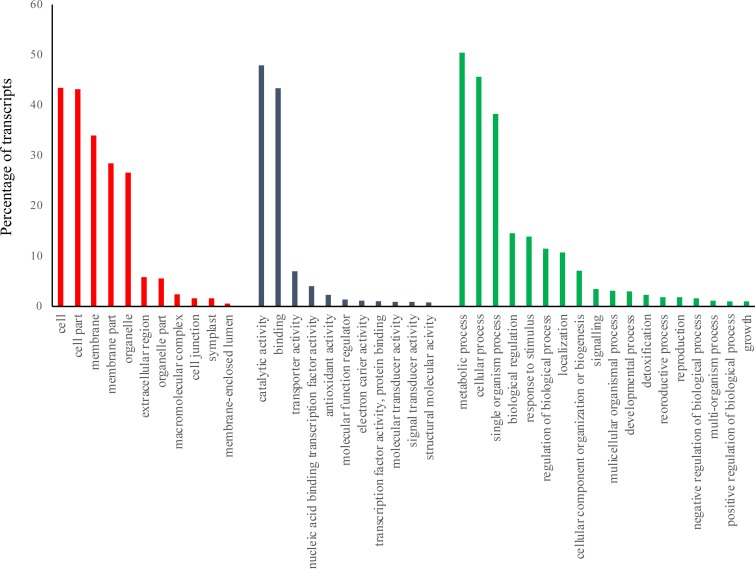
GO classification of DEGs based on sequence homology to 3 main categories of cellular component, molecular function and biological process.

The dominant terms were ‘cell’, ‘cell part’ and ‘membrane’ in cellular component category while ‘catalytic activity’ and ‘binding’ were the most dominant terms in molecular function category. In biological process category, most of the DE genes were classified in metabolic process and cellular process followed by single organism process, biological regulation and response to stimulus (Fig 2). The transcriptional changes of genes categorized in metabolic and cellular processes were previously reported under different environmental conditions[63, 64] proposing that extensive metabolic activities occur in the stress treated plants.

Not surprisingly, the most enriched biological process terms for over-presented DEGs were electron transport, response to oxidative stress, response to chemical stimulus, carbohydrate metabolic process, plant-type cell-wall organization, transport and establishment of localization which acted as indicators of significant biological processes underlying the specific salinity-stress responses of plants and are in agreement with those reported in salt stress response in previous studies[12, 28, 65].The most enriched molecular function terms for over-presented DEGs were catalytic activity, iron ion binding, tetrapyrrole binding, cation binding, oxidoreductase activity and antioxidant activity. Meanwhile, with regard to the over-representation of cellular component terms among the DEGs, we found extracellular region, membrane, intrinsic to membrane, integral to membrane and intrinsic to plasma membrane to be the most enriched (S10 Table).

### Functional annotation and classification of novel DEGs

Blast2GO was used to compare the functional annotation of novel DEGs against the NCBI non redundant (nr) protein database with a cut-off E-value of 1.0 E^−3^. Of the 544 novel DEGs, 405 genes (74.4%) aligned to nr protein database whereas the remaining 139 genes (25.6%) did not show homology to any sequence in the database. Sequence homology based on GO categorization utilizing Blast2GO tool indicated that out of all the novel DEGs, 259 genes (47.6%) were assigned GO terms and 229 genes (42%) were classified in significant GO terms (S2A Fig). In biological process category, the majority of genes were engaged in metabolic process and cellular process followed by single-organism process, localization, response to stimulus and biological regulation. With respect to the cellular component, cell part, cell and membrane were the dominant groups, followed by membrane part, organelle, organelle part and extracellular region. With regard to molecular function, the top three categories were catalytic activity, binding and transporter activity (S2B Fig).

It is expected that some of the novel DEGs play potential roles in salt stress tolerance such as the genes coding for peroxidase[66], Glutathione S-trasferase[67], Phosphatase 2C[68], Na^+^/Ca^2+^ Exchanger-like Protein[38], pathogenesis-related protein[69], salt stress-induced hydrophobic peptide ESI3(Early Salt stress Induced 3)[70], ATP synthase subunit beta[71], Late Embryogenesis Abundant protein[72], WRKY[73], MADS-box[74] and bHLH transcription factors[75] and cytochrome P450 monooxygenase[76] (S11 Table).

### KEGG pathway classification of DEGs

**Fig 3:**
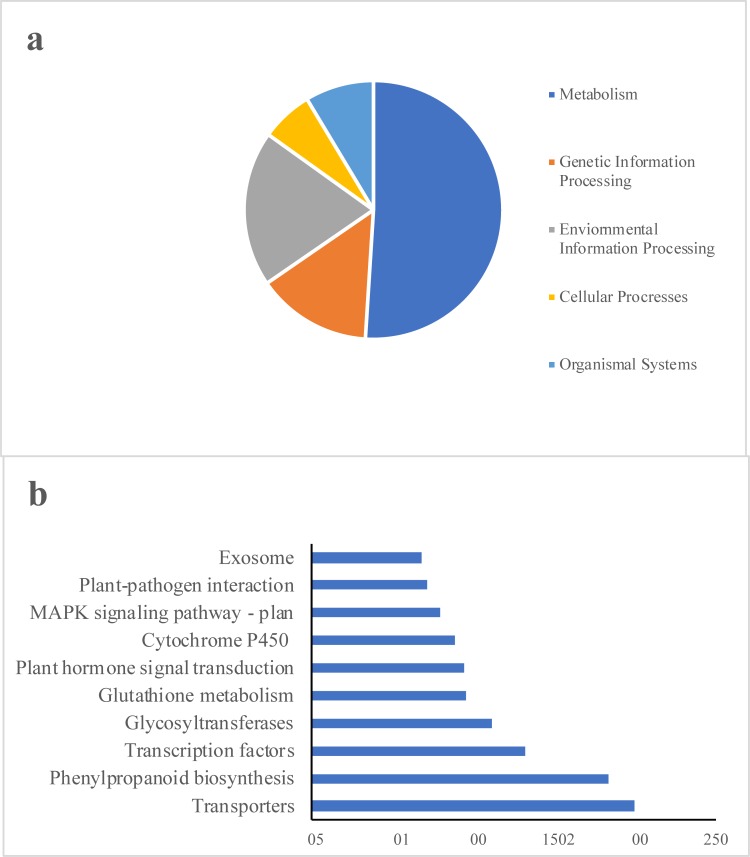
KEGG classification of the DEGs. (a) KEGG distribution of the annotated genes into 5 main categories.(b) The top 10 pathways with the highest number of genes.

For a better understanding of the active biological pathways in the DEGs under salt stress, a single-directional BLAST search against KEGG protein database was done using KAAS server[23, 77]. This is a method to classify gene functions with emphasis on the biochemical pathways. The results indicated that 1744 of the 5128 DEGs could be classified into 227 KEGG pathways, covering the five main KEGG categories of metabolism, environmental information processing, genetic information processing, organismal systems and cellular processes (Fig 3A). These genes belonged mainly to the following KEGG pathways: Transporters, phenylpropanoid biosynthesis, transcription factors, glycosyltransferases, glutathione metabolism, plant hormone signal transduction, cytochrome P450, MAPK signaling pathway–plant, plant-pathogen interaction and exosome (Fig 3B and S12 Table) that are significant pathways in abiotic and biotic stress response in plants and have been reported in previous studies[12, 65, 78]. Phenylpropanoid pathway with the second highest number of genes is a metabolic pathway liable for the synthesis of different plant secondary metabolites having roles in developmental and stress–related processes[79]. In this study, genes such as peroxidases, shikimate O-hydroxycinnamoyl transferases, caffeic acid 3-O-methyltransferases and beta-glucosidases were up-regulated in this pathway. Beta-Glucosidases are known to play a role in abiotic stresses via accumulation of reactive oxygen species (ROS) scavenging flavonols[80]. Plants use accumulation of lignin or alteration of the monomeric composition of lignin in the cell wall to overcome salt stress[81]. Up-regulation of shikimate hydroxycinnamoyl transferase and caffeic acid 3-O-methyltransferase, both engaged in lignification, have been reported under salt stress in previous studies [82, 83].

### Alternative splicing analysis

Alternative splicing (AS) is a fundamental molecular mechanism increasing transcriptome and proteome complexity and diversity in higher eukaryotes. It is reported that alternative splicing is involved in a range of functions in plants, such as growth, development, signal transduction, and responses to biotic and abiotic stress[84–89].

In this study, 37% of the multi exonic genes were alternatively spliced at the whole transcriptome level. Alternative splicing has been reported in 61% of *Arabidopsis thaliana* genes and 21.2 to 33% of rice (*Oryza sativa*) genes[90, 91]. Our results revealed that 120668 alternative splicing events occurred in our assembly, of which the highest number belonged to intron retention (IR) with 43719 (36%) events, followed by alternate 3 acceptor (AA), alternate 5 donor (AD) and exon skipping (ES) represented by 20578 (17%), 13119 (11%) and 8124 (7%) events, respectively. Intron retention was the most dominant alternative splicing event in our assembly which was in line with the studies in other plants such as sorghum, rice, brachypodium and Arabidopsis.

Functional categorization of the transcripts created via IR AS event indicated their involvement in biological processes such as metabolic process, regulation of biological process, response to stimulus, localization, cellular component organization, developmental process and signaling (S3 Fig).

In addition, alternative splicing analysis was performed on control and salt-treated samples separately. Results showed that 89456 and 87606 alternative splicing events were obtained in salt-treated samples while 86882 and 86721 alternative splicing events were observed in control samples. Therefore, there is an increase in alternate splicing frequency under salt stress conditions, in accordance with the relevant report in Arabidopsis (S4 Fig). [92]. This indicates on the possible roles of AS in plant response to salt stress.

### Alternative splicing in DEGs

**Fig 4:**
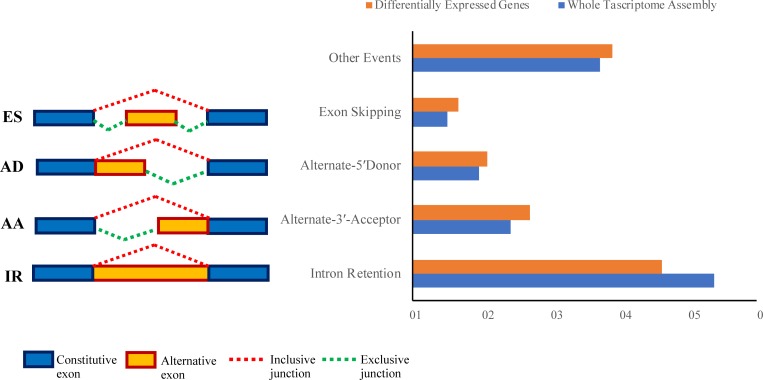
Analysis of alternative splicing. Bar chart showing the percentage of different types of alternative splicing events at the whole trascriptome assembly and differentially expressed genes AD: Alternate-5′Donor; AA: Alternate-3′-Acceptor, IR: Intron Retention and ES: Exon Skipping.

In this study, we identified 3884 alternative splicing events in the DEGs under salt stress. In total, 30% of DEGs (1482 genes out of 5128 genes) were alternatively spliced and produced 4041 different isoforms. Among the different alternative splicing types, intron retention events predominated (1706), followed by AA (552), AD (372) and ES (197) events. In addition, 1057 events were classified as complex alternative splicing events. The ratio of different AS events in DEGs was similar to the ratio of different AS events at the whole transcriptome assembly (Fig 4).

Functional categorization of the alternatively spliced DEGs revealed their involvement in different pathways related to stress responses. Genes related to the biological processes of transport, nucleic acid metabolic processes, localization, response to stimulus, response to stress, metal ion transport, response to oxidative stress, signaling process, regulation of RNA metabolic process and transcription were enriched in the alternatively spliced DEGs (S5 Fig). We discovered alternative splicing events in many important genes engaged in salt stress response such as CBL-interacting kinases, serine-threonine kinases, different transcription factors (MYBs, NACs, bZIPs, HSTFs and WRKYs), phosphatases 2c, peroxidases, Gluthatione S-transferases, LEA proteins, Calcium transporting ATPases, Calcium binding proteins, antiporters, salt tolerant-related proteins, salt response proteins etc. (S13 Table).

### Metabolic pathways involved in salt stress response

**Fig 5:**
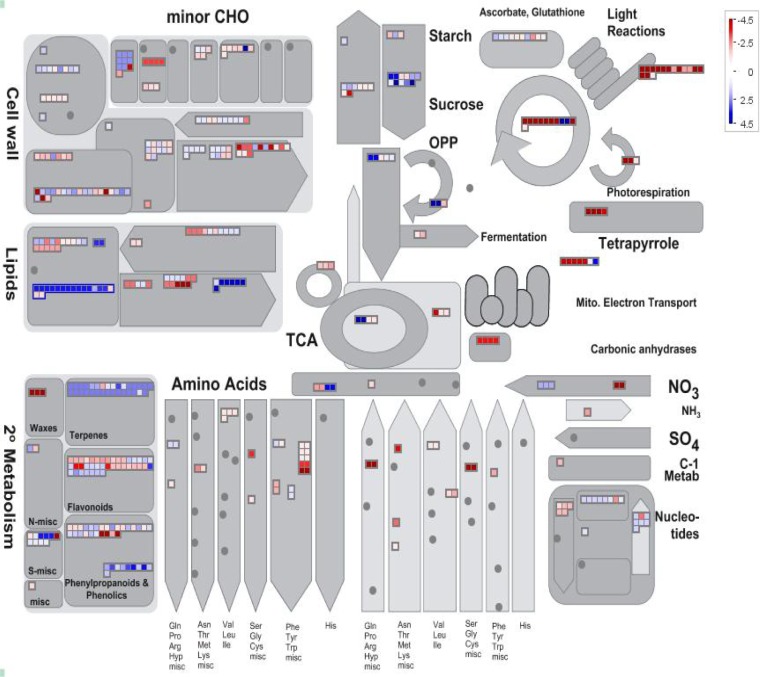
Metabolic pathways overview of DEGs in *T*.*aestivum* under salt stress using Mapman. blue: up-regulated genes and red: down-regulated genes.

As a complementary approach to the GO analysis, we searched the putative functions of the salt responsive genes identified using MapMan[24], allowing the visualization of salt induced changes in different metabolic processes. The results of mapping the DEGs to the metabolic pathway overview revealed that lipid and sucrose metabolism pathways were among the enriched pathways (Fig 5 and S14 Table). In sucrose metabolism pathway, genes encoding for hexokinase, cell wall invertase and sucrose synthase are up-regulated under salt stress. Abiotic stresses usually lead to sugar accumulation [93]. Acumulation of glucose, sucrose and fructose under high salinity plays a key role in carbon storage, osmotic regulation, homeostasis and ROS scavenging[94]. Sun et al.[62] reported that exogenous application of glucose enhanced salt tolerance in apple and hexokinase1, acting as glucose sensor, contributed to Glc-mediated salinity tolerance. Hexokinase1 interacted with Tonoplast-localized Na^+^/H^+^ exchanger (NHX1) and phosphorylated it. Phosphorylation improved the stability of Na^+^/H^+^ exchanger and enhanced its Na^+^/H^+^ transport activity in transgenic apple overexpressing NHX1. Overexpression of the cell wall invertase gene from *Chenopodium rubrum* improved drought tolerance in tomato. This gene critically acts at the integration point of metabolic, hormonal, and stress signals, preparing a novel strategy to conquer drought-induced limitations to crop yield[95].

The cellular overview pathway showed that genes coding for abiotic stress related miscellaneous enzyme families (misc) and glutaredoxins were up-regulated in *T*. *aestivum* under salinity stress (S6 Fig and S14 Table). Glutaredoxins (GRXs) are small redox proteins, which use glutathione to catalyze the reduction of disulfide bonds of substrate proteins to maintain cellular redox homeostasis. Furthermore, diverse functions such as transcriptional regulation of defense responses, flower development, oxidative stress response, redox signaling, hormonal regulation, iron homeostasis, and environmental adaptation have been reported for various plant GRXs[96].

The secondary metabolite pathway overview revealed that the genes involved in terpenoid, lignin, phenols and isoflavonoid metabolic pathways were significantly enriched in this salinity tolerant bread wheat variety under salinity stress (S7 Fig and S14 Table). Terpenoids are the largest and most diverse group of chemicals produced by plants. Plants employ terpenoids in basic functions such as growth and development but the majority of terpenoids are used for protection against the abiotic and biotic stresses. Increase in the expression of genes encoding terpenoids and their role in coping with salt stress have been reported in Mangrove plants[97].

In addition, the stress response pathways indicated that the genes involved in ethylene signalling pathways and genes coding for transcription regulators and peroxidases were found to be enriched in *T*. *aestivum* under salinity stress (S8 Fig and S14 Table).

### Validation of differential gene expression using qRT-PCR

**Fig 6:**
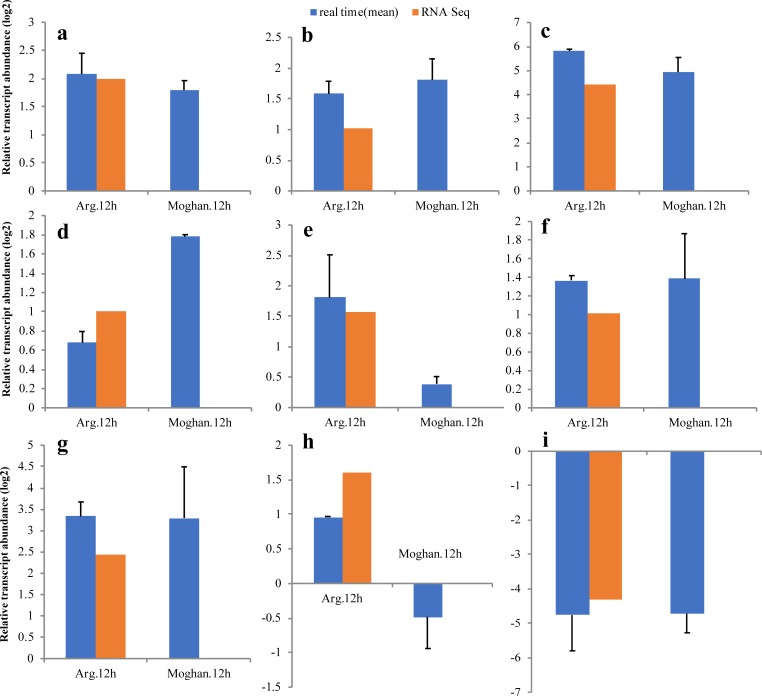
Validation of selected genes using qRT-PCR. (a) Calcineurin B-like protein (CBL)-CBL-interacting protein kinase 31 (*Ta*.*CIPK31*); (b) NAC transcription factor 15 (*Ta*. *NAC15*); (c) Heat Shock transcription factor C1b (*Ta*. *HsfC1b*);(d) Salt Overly Sensitive 1 (*Ta*. *SOS1*); (e) Dehydration-responsive protein RD22 (*Ta*. *RD22*) (f) bHLH transcription factor (*Ta*. *bHLH*); (g) late embryogenesis abundant protein 3 (*Ta*. *LEA3*); (h) salt tolerant-related protein (*Ta*. *STRP*); (i) Proline oxidase (*Ta*. *POX*). The gene ensemble Ids are mentioned in the S1 Table.

To further validate the RNA-Seq expression profiling, nine salt responsive genes were selected for qRT-PCR (Fig 6). The qRT-PCR results were highly consistent with those of RNA sequencing (R^2^ = 0.98). Therefore, the DEGs identified in this study can be considered to have a high accuracy. To achieve further insight, the expression pattern of these genes was compared between two cultivars. The results of qRT-PCR analysis showed that there was no significant difference in the expression of the selected genes between Arg and Moghan3 cultivars or weaker response was observed in the susceptible (Moghan3) cultivar except for SOS1 which showed higher expression in Moghan3 (Fig 6). It is probable that the sensitive genotype has not been able to reduce the sodium level using other approaches, so up-regulation of this antiporter might diminish the sodium level via sodium excretion.

In summary, this study prepares a comprehensive overview of the transcriptome changes of an Iranian salt tolerant bread wheat cultivar under salt stress. Using TGACv1 reference genome of the bread wheat for analysis, more than 85% of the total reads were mapped to this reference genome. Moreover, around 26171 novel transcripts were identified which can improve the genome annotation of *T*. *aestivum*. Multiple genes and several key pathways were recognized to be involved in salt tolerance. In addition, 3884 alternative splicing events were identified in the salt responsive genes and IR was the most dominant event. Overall, the achieved results could improve the current understanding of salt stress response in root tissue of bread wheat, but further research studies will be required to examine the application of the detected genes as biomarkers for marker-assisted breeding or cadidates for genetic engineering in order to obtain salt tolerant plants.

## Conclusion

**Fig 7:**
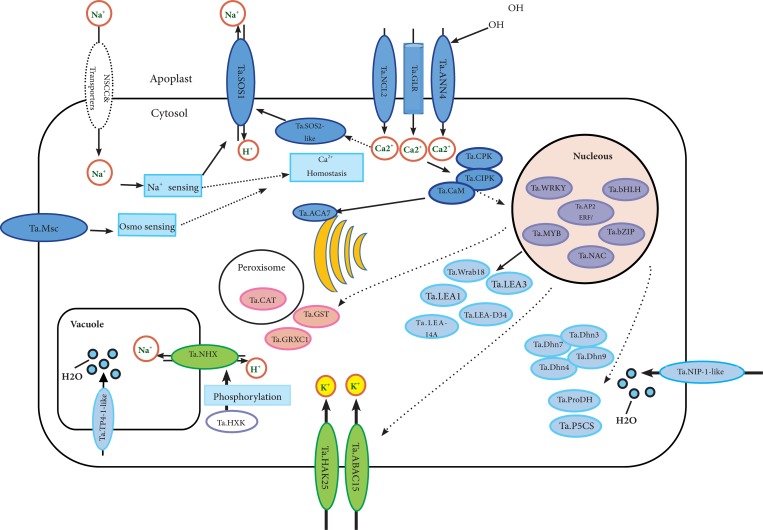
Model proposed for salt stress response in Arg. Sensing and signaling components and transcription factors highlighted in dark blue and purple, respectively. Mechanisms engaged in response to osmotic, ionic and oxidative stresses arising from salinity depicted in light blue, light green and pink, respectively.

This study presents a comprehensive overview of the transcriptome changes of an Iranian salt tolerant bread wheat cultivar, Arg, under salt stress, which can help understanding the molecular basis of salinity tolerance in *T*. *aestivum*. A model is proposed for salt stress response in Arg cultivar employing the DEGs (Fig 7) (S15 Table and. S9 Fig). Based on the achieved results, salinity-induced osmotic and ionic stress might be sensed by mechanosensitive ion channels (e.g. Ta.Msc) and membrane Na^+^/H^+^ antiporter (Ta.SOS1), respectively. After sensing the stress, signaling cascades are triggered[6]. To this end, Ca^+2^ has been reported to serve as a secondary messenger, so an increase in cytosolic Ca^+2^ concentrations is expected[98]. In this study, the genes coding for Ca^2+^ transporters such as *Ta*.*ANN4*, *Ta*.*ACA7* and *Ta*.*NCL2* were appeared to be up-regulated, which may adjust the Ca^+2^ cytosolic concentrations. *Ta*.*GLR*, which encodes a non-selective cation channel were also induced in Arg under salt stress, and is supposed to be involved in Ca^2+^ transport. The genes coding for CaM, CIPK and CPK were also up-regulated, which are involved in Ca^+2^ signaling pathway [41, 42, 46]. The genes coding for transcription factors such as MYB, NAC, bHLH, WRKY, bZIPs and AP2/ERF were observed among the DEGs. Some of these genes have been proved to be involved in salt stress response based on the information about their orthologues in Arabidopsis (S1 Table). These transcription factors can regulate the expression of the genes engaged in dealing with osmotic, ionic and oxidative stresses arising from salinity[6]. The genes coding for Aquaporins (*Ta*.*TP4-1-like* and *Ta*.*NIP1-1-like*), LEA proteins (*Ta*.*Wrab18*, *Ta*.*LEA1*, *Ta*.*LEA3*, *Ta*.*LEA-D34-Like* and *Ta*.*LEA14*-A) and dehydrins (*Ta*.*DHN3*, *Ta*.*DHN4*, *Ta*.*DHN7* and *Ta*.*DHN9*), P5CS (involved in proline synthesis) (*Ta*.*P5CS*) with increased expression and proline oxidase (*Ta*.*ProDH*) (involved in proline degradation) with decreased expression can alleviate the osmotic stress. In order to deal with the ionic stress, plasma membrane Na^+^/H^+^ antiporter SOS1, K^+^ transporters (such as *Ta*.*HAK25*) and ABC transporters (such as *Ta*.*ABAC15*) were significantly up-regulated under salt stress. The gene coding for SOS2-like protein kinase PKS12 is likely to control the activity of the Na^+^/H^+^ antiporter SOS1. Although the transcript level of Na^+^/H^+^ antiporter NHX1 was not increased under salinity in this study, but the protein encoded by up-regulated *Ta*.*HXK1* is able to phosphorylate Ta-NHX1, leading to higher compartmentalization of Na^+^ into vacuole. In addition, the genes coding for catalases (*Ta*.*CAT*), glutaredoxins (*Ta*.*GRXC1*) and Gluthatione-S- transferases (*Ta*.*GST*) appear to deal with oxidative stress (Fig 7). We hope the attained results could be useful toward achieving salt tolerant cultivars through molecular breeding or genetic engineering.

## Supporting information

S1 Fig Hierachical clustering for all DEGs using Heatmapper online software (http://www.heatmapper.ca). Each row reperesents a separate gene expression and each column a separate mRNA sample.
(DOCX)

S2 Fig (a) Annotation statistics of novel DEGs. (b) GO annotation clusters of novel differentially expressed genes between normal and salt stress.
(DOCX)

S3 Fig GOSlim terms (in biological process category) assigned to the transcripts produced through IR AS event. (DOCX)

S4 Fig Alternavive splicing events happened in normal and salt-treated samples. (DOCX)

S5 Fig Gene ontology enrichment for biological process category for the differentially expressed genes having alternative splicing events. (DOCX)

S6 Fig Cellular response pathways overview of differentially expressed genes in *Triticum aestivum* under salt stress. blue,up-regulated genes and red,down-regulated genes.
(DOCX)

S7 Fig Secondary metabolic pathways overview of differentially expressed genes in *Triticum aestivum* under salinity stress. blue,up-regulated genes and red,down-regulated genes.
(DOCX)

S8 Fig Biotic stress pathways overview of differentially expressed genes in *Triticum aestivum* under salinity stress. blue,up-regulated genes and red,down-regulated genes.
(DOCX)

S9 Fig Hierachical clustering for DEGs located in the proposed model using Heatmapper online software ((http://www.heatmapper.ca). (DOCX)

S1 Table Primers used in the study. (DOCX)

S2 Table Summary of sequencing results. (DOCX)

S3 Table The percentage of annotated and unannotated known and novel transcripts. (XLSX)

S4 Table Biological process terms assigned to novel transcripts. (XLSX)

S5 Table Molecular function terms assigned to novel transcripts. (XLSX)

S6 Table Cellular component terms assigned to novel transcripts. (XLSX)

S7 Table List of transcripts exclusively expressed under salt stress. (XLSX)

S8 Table List of differentially expressed genes. (XLSX)

S9 Table List of some published salt responsive genes among the DEGs detected by RNA-Seq. (XLS)

S10 Table Gene ontology terms assigned to differentially expresssed genes. (XLSX)

S11 Table List of novel differentially expressed genes. (XLSX)

S12 Table KEGG pathway classification of DEGs. (XLSX)

S13 Table List of differentially expressed genes having alternative splicing events. (XLSX)

S14 Table The results of mapping the DEGs to the metabolic pathway overview. (DOCX)

S15 Table List of some salt responsive genes located in the proposed model. (DOCX)
