# Supplementary material for: Transcriptome response of roots to salt stress in a salinity-tolerant bread wheat cultivar
Source: PLoS One. 2019 Mar 15;14(3):e0213305. doi: 10.1371/journal.pone.0213305 (PMC6420002; doi:10.1371/journal.pone.0213305)
Supplement: S8 Fig — blue,up-regulated genes and red,down-regulated genes. (DOCX) [file pone.0213305.s008.docx]

**RNA-Seq analysis of Bread Wheat Root Transcriptome in Response to Salt Stress**

**Functional and Integrative Genomics**

N. Amirbakhtiar^1^, A. Ismaili^1^*, M.R. Ghaffari^2^, F. Nazarian Firouzabadi^1^, Z.S. Shobbar^2^*

1- Department of Agronomy and Plant Breeding, Faculty of Agriculture, Lorestan University, PO Box 465, Khorramabad, Iran.

2- Department of Systems Biology, Agricultural Biotechnology Research Institute of Iran (ABRII), Agricultural Research, Education and Extension Organization (AREEO), PO Box 31535-1897, Karaj, Iran

* Co-corresponding authors:

Zahra-Sadat Shobbar: Email: [shobbar@abrii.ac.ir](mailto:shobbar@abrii.ac.ir); Phone: +98-2632703536. Ahmad Ismaili: Email: ismaili.a@lu.ac.ir; Phone: +98-66-33400012.


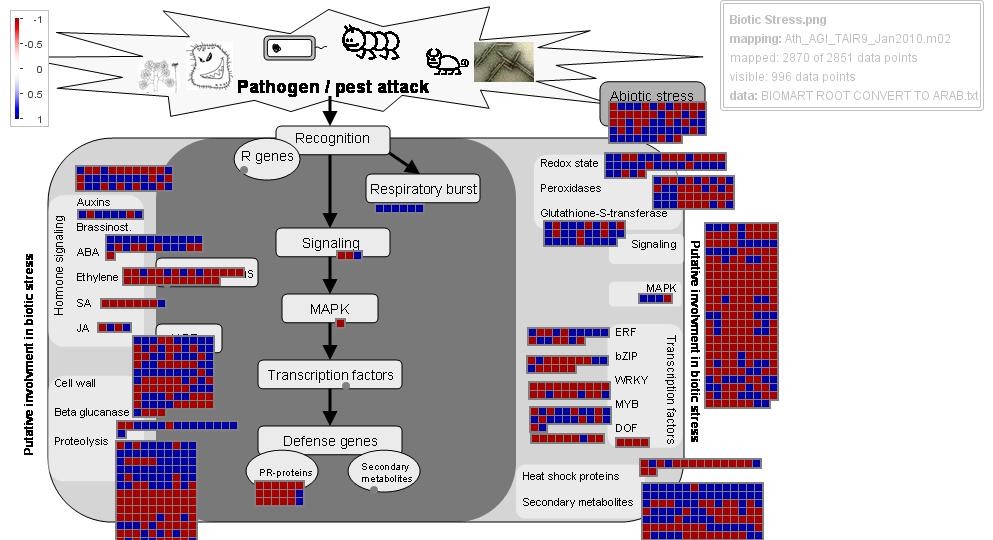


S8 Fig.. Biotic stress pathways overview of differentially expressed genes in *Triticum* *aestivum* under salinity stress. blue,up-regulated genes and red,down-regulated genes.
