# Supplementary material for: Transcriptome response of roots to salt stress in a salinity-tolerant bread wheat cultivar
Source: PLoS One. 2019 Mar 15;14(3):e0213305. doi: 10.1371/journal.pone.0213305 (PMC6420002; doi:10.1371/journal.pone.0213305)
Supplement: S9 Fig — (DOCX) [file pone.0213305.s009.docx]

**RNA-Seq analysis of Bread Wheat Root Transcriptome in Response to Salt Stress**

**Functional and Integrative Genomics**

N. Amirbakhtiar^1^, A. Ismaili^1^*, M.R. Ghaffari^2^, F. Nazarian Firouzabadi^1^, Z.S. Shobbar^2^*

1- Department of Agronomy and Plant Breeding, Faculty of Agriculture, Lorestan University, PO Box 465, Khorramabad, Iran.

2- Department of Systems Biology, Agricultural Biotechnology Research Institute of Iran (ABRII), Agricultural Research, Education and Extension Organization (AREEO), PO Box 31535-1897, Karaj, Iran

* Co-corresponding authors:

Zahra-Sadat Shobbar: Email: [shobbar@abrii.ac.ir](mailto:shobbar@abrii.ac.ir); Phone: +98-2632703536. Ahmad Ismaili: Email: ismaili.a@lu.ac.ir; Phone: +98-66-33400012.


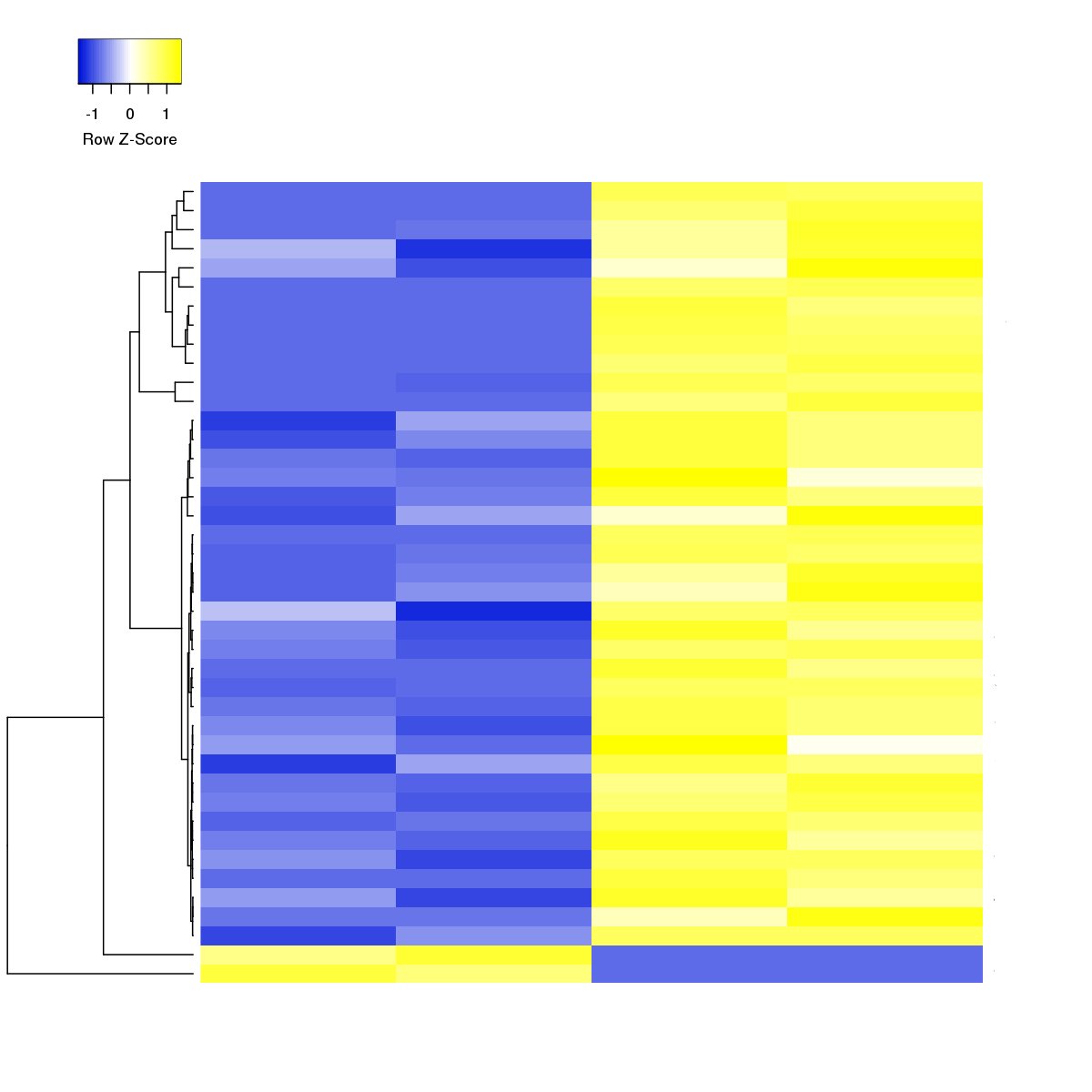


Ta.HAK25

Ta.Dhn4

Ta.Wrab18

Ta.Lea14-A-like

Ta.LEA1

Ta.Dhn4

Dehydrin- LEA group 2

Ta.DHN3

Ta.DHN3

Ta.DHN3

Ta.DHN3

Ta.DHN7

Ta.POX

Ta.ANN4

Ta.Dhn9

Ta.POX

Ta.LEA14-A

Ta.POX

Ta.P5CS

Ta.P5CS

Ta.P5CS

Ta.POX

Ta.SOS1

Ta.CAT

Ta.POX

Ta.LEA-D34-Like

Ta.Dhn9

Dehydrin- LEA group 2

Ta.HXK1

Ta.POX

Ta.Msc6

Ta.NIP1-1-like

Ta.POX

Ta.SOS2-like

Ta.POX

Ta.POX

Ta.LEA-D34-Like

Ta.GLR

Ta.GRXC1

Ta.Msc6

Ta.ProDH

Ta.ProDH

S9 Fig. Hierachical clustering for DEGs located in the proposed model using Heatmapper online software (http://www.heatmapper.ca).

**Normal Replication2**

**Salt-treated Replication2**

**Salt-treated Replication1**

**Normal Replication1**
