# Supplementary material for: Transcriptome response of roots to salt stress in a salinity-tolerant bread wheat cultivar
Source: PLoS One. 2019 Mar 15;14(3):e0213305. doi: 10.1371/journal.pone.0213305 (PMC6420002; doi:10.1371/journal.pone.0213305)
Supplement: S2 Table — (DOCX) [file pone.0213305.s011.docx]

**RNA-Seq analysis of Bread Wheat Root Transcriptome in Response to Salt Stress**

**Functional and Integrative Genomics**

N. Amirbakhtiar^1^, A. Ismaili^1^*, M.R. Ghaffari^2^, F. Nazarian Firouzabadi^1^, Z.S. Shobbar^2^*

1- Department of Agronomy and Plant Breeding, Faculty of Agriculture, Lorestan University, PO Box 465, Khorramabad, Iran.

2- Department of Systems Biology, Agricultural Biotechnology Research Institute of Iran (ABRII), Agricultural Research, Education and Extension Organization (AREEO), PO Box 31535-1897, Karaj, Iran

* Co-corresponding authors:

Zahra-Sadat Shobbar: Email: [shobbar@abrii.ac.ir](mailto:shobbar@abrii.ac.ir); Phone: +98-2632703536. Ahmad Ismaili: Email: ismaili.a@lu.ac.ir; Phone: +98-66-33400012.

S2 Table. Summary of sequencing results

| **Sample name** | **Raw reads** (paired end) | **Clean reads** (paired end) | **Q20%** | **Q30%** |
| --- | --- | --- | --- | --- |
| Control-rep1 | 27,683,639 | 27,525,982 | 96.98 | 92.53 |
| Control-rep2 | 25,913,048 | 25,444,146 | 96.48 | 91.43 |
| Salt-stressed-rep1 | 30,470,822 | 29,966,827 | 96.32 | 91.30 |
| Salt-stressed-rep2 | 29,278,109 | 28,799,611 | 96.44 | 91.49 |
| Total | 113,345,618 | 111,736,566 | ≥ 96.32 | ≥ 91.30 |
