# Supplementary material for: Transcriptome response of roots to salt stress in a salinity-tolerant bread wheat cultivar
Source: PLoS One. 2019 Mar 15;14(3):e0213305. doi: 10.1371/journal.pone.0213305 (PMC6420002; doi:10.1371/journal.pone.0213305)
Supplement: S14 Table — (DOCX) [file pone.0213305.s023.docx]

**RNA-Seq analysis of Bread Wheat Root Transcriptome in Response to Salt Stress**

**Functional and Integrative Genomics**

N. Amirbakhtiar^1^, A. Ismaili^1^*, M.R. Ghaffari^2^, F. Nazarian Firouzabadi^1^, Z.S. Shobbar^2^*

1- Department of Agronomy and Plant Breeding, Faculty of Agriculture, Lorestan University, PO Box 465, Khorramabad, Iran.

2- Department of Systems Biology, Agricultural Biotechnology Research Institute of Iran (ABRII), Agricultural Research, Education and Extension Organization (AREEO), PO Box 31535-1897, Karaj, Iran

* Co-corresponding authors:

Zahra-Sadat Shobbar: Email: [shobbar@abrii.ac.ir](mailto:shobbar@abrii.ac.ir); Phone: +98-2632703536. Ahmad Ismaili: Email: ismaili.a@lu.ac.ir; Phone: +98-66-33400012.

| Table S14. List of some published salt responsive genes among the DEGs detected by RNA-Seq. | | | | |
| --- | --- | --- | --- | --- |
| **Wheat gene ID** | **Arabidopsis orthologue** | **Gene name** | **Log2(FC)** | **References** |
| TRIAE_CS42_4BL_TGACv1_321295_AA1058570 | AT2G26980 | CIPK3 | 2.4 | (Kim et al., 2003)^39^ |
| TRIAE_CS42_4DL_TGACv1_342497_AA1115230 | AT2G26980 | CIPK3 | 1.4 | (Kim et al., 2003)^39^ |
| TRIAE_CS42_1BS_TGACv1_050098_AA0167360 | AT3G17510 | CIPK1 | 1.01 | (Albrecht et al., 2006)^38^ |
| TRIAE_CS42_5AL_TGACv1_375277_AA1218980 | AT1G01140 | CIPK9 | -1.3 | (Pandey et al., 2007)^40^ |
| TRIAE_CS42_U_TGACv1_644157_AA2137680 | AT1G01140 | CIPK9 | -1.03 | (Pandey et al., 2007)^40^ |
| TRIAE_CS42_4BL_TGACv1_322341_AA1070740 | AT1G01140 | CIPK9 | -1.4 | (Pandey et al., 2007)^40^ |
| TRIAE_CS42_2AL_TGACv1_093319_AA0277330 | AT3G29035 | NAC59 | 1.6 | (Balazadeh et al. 2011)^98^ |
| TRIAE_CS42_2DL_TGACv1_158215_AA0512750 | AT5G39610 | NAC92 | 1.8 | (He et al. 2005)^44^ |
| TRIAE_CS42_1BL_TGACv1_032010_AA0124150 | AT1G77450 | NAC032 | 1.1 | (Mahmood, et al. 2016)^46^ |
| TRIAE_CS42_3B_TGACv1_221306_AA0736740 | - | NAC protein | 1.3 | (Kawaura et al. 2008)^45^ |
| TRIAE_CS42_3AL_TGACv1_196305_AA0659290 | AT2G40140 | SZF2 | -1.1 | (Sun et al., 2007)^21^ |
| TRIAE_CS42_5AS_TGACv1_393865_AA1276600 | AT5G43170 | AZF3 | -1.7 | (Sakamoto et al., 2004)^99^ |
| TRIAE_CS42_5DL_TGACv1_433002_AA1397630 | AT5G43170 | AZF3 | -1.2 | (Sakamoto et al., 2004)^99^ |
| TRIAE_CS42_5BS_TGACv1_423181_AA1369440 | AT5G43170 | AZF3 | -1.1 | (Sakamoto et al., 2004)^99^ |
| TRIAE_CS42_7DS_TGACv1_622616_AA2042680 | AT1G27730 | ZAT10 | -1.7 | (Mittler et al., 2006)^100^ |
| TRIAE_CS42_7AS_TGACv1_569571_AA1819320 | AT1G27730 | ZAT10 | -1.6 | (Mittler et al., 2006)^100^ |
| TRIAE_CS42_U_TGACv1_640742_AA2072200 | AT1G27730 | ZAT10 | -1.9 | (Mittler et al., 2006)^100^ |
| TRIAE_CS42_3AL_TGACv1_196305_AA0659290 | AT3G55980 | SZF1 | -1.1 | (Sun et al., 2007)^21^ |
| TRIAE_CS42_2DS_TGACv1_177763_AA0584170 | AT4G05100 | MYB74 | 2.7 | (Xu et al., 2015)^101^ |
| TRIAE_CS42_2AS_TGACv1_112690_AA0343530 | AT4G28110 | MYB41 | 2.3 | (Hoang et al., 2012)^102^ |
| TRIAE_CS42_7AS_TGACv1_570884_AA1842010 | - | TaMYBsdu1 | 22.4 | (Rahaie et al., 2010)^103^ |
