# Supplementary material for: Transcriptome response of roots to salt stress in a salinity-tolerant bread wheat cultivar
Source: PLoS One. 2019 Mar 15;14(3):e0213305. doi: 10.1371/journal.pone.0213305 (PMC6420002; doi:10.1371/journal.pone.0213305)
Supplement: S15 Table — (DOCX) [file pone.0213305.s024.docx]

**RNA-Seq analysis of Bread Wheat Root Transcriptome in Response to Salt Stress**

**Functional and Integrative Genomics**

N. Amirbakhtiar^1^, A. Ismaili^1^*, M.R. Ghaffari^2^, F. Nazarian Firouzabadi^1^, Z.S. Shobbar^2^*

1- Department of Agronomy and Plant Breeding, Faculty of Agriculture, Lorestan University, PO Box 465, Khorramabad, Iran.

2- Department of Systems Biology, Agricultural Biotechnology Research Institute of Iran (ABRII), Agricultural Research, Education and Extension Organization (AREEO), PO Box 31535-1897, Karaj, Iran

* Co-corresponding authors:

Zahra-Sadat Shobbar: Email: [shobbar@abrii.ac.ir](mailto:shobbar@abrii.ac.ir); Phone: +98-2632703536. Ahmad Ismaili: Email: ismaili.a@lu.ac.ir; Phone: +98-66-33400012.

| S15 Table. List of some salt responsive genes located in the proposed model | | | | |
| --- | --- | --- | --- | --- |
| **Row number** | **Ensembl Id/Location** | **Log(FC)** | **Description** | **Gene name** |
| 1 | TRIAE_CS42_6AL_TGACv1_471216_AA1504800 | 1.28181 | Mechanosensitive ion channel 6 | **Ta.Msc6** |
| 2 | TRIAE_CS42_6BL_TGACv1_501742_AA1620350 | 2.76352 | Mechanosensitive ion channel 6 | **Ta.Msc6** |
| 3 | TRIAE_CS42_6DL_TGACv1_527735_AA1708050 | 1.42691 | Mechanosensitive ion channel 6 | **Ta.Msc6** |
| 4 | TRIAE_CS42_3DS_TGACv1_271660_AA0905010 | 1.1 | salt overly sensitive 1 | **Ta.SOS1** |
| 6 | TRIAE_CS42_1AL_TGACv1_000627_AA0016200 | 1.4 | Annexin D4 | **Ta.ANN4** |
| 7 | Novel gene(TGACv1_scaffold_641741_U:17189-17824) | 3.4 | Calcium-transporting ATPase plasma membrane-type | **Ta.ACA** |
| 8 | Novel gene(TGACv1_scaffold_571144_7AS:15506-18943) | 3.4 | Sodium/calcium exchanger | **Ta.NCL2** |
| 9 | TRIAE_CS42_3B_TGACv1_223131_AA0776870 | 2.6 | aquaporin NIP1-1-like | **Ta.NIP1-1-like** |
| 10 | TRIAE_CS42_4BL_TGACv1_320425_AA1038680 | 1.1 | Putative aquaporin TIP4-1-like | **Ta.TP4-1-like** |
| 11 | TRIAE_CS42_6BL_TGACv1_503721_AA1628330 | 8.2 | dehydrin 7 | **Ta.DHN7** |
| 12 | TRIAE_CS42_6DL_TGACv1_529405_AA1718250 | inf | dehydrin 7 | **Ta.DHN7** |
| 13 | TRIAE_CS42_6AL_TGACv1_473324_AA1530160 | 5.4 | DHN3 | **Ta.DHN3** |
| 14 | TRIAE_CS42_6AL_TGACv1_473324_AA1530170 | 5.6 | DHN3 | **Ta.DHN3** |
| 15 | TRIAE_CS42_6BL_TGACv1_503808_AA1628460 | 6.1 | DHN3 | **Ta.DHN3** |
| 16 | TRIAE_CS42_6DL_TGACv1_526490_AA1685170 | 3.7 | DHN3 | **Ta.DHN3** |
| 17 | TRIAE_CS42_6DL_TGACv1_527031_AA1697450 | 4.5 | DHN3 | **Ta.DHN3** |
| 18 | TRIAE_CS42_7AL_TGACv1_556589_AA1766430 | 5.8 | dehydrin- LEA group 2 | - |
| 19 | TRIAE_CS42_7BL_TGACv1_578284_AA1892310 | 2.5 | dehydrin- LEA group 2 | - |
| 20 | TRIAE_CS42_7DL_TGACv1_602743_AA1967200 | 4.2 | dehydrin- LEA group 2 | - |
| 21 | TRIAE_CS42_6BL_TGACv1_500727_AA1608920 | 6.1 | Dhn4 | **Ta.Dhn4** |
| 22 | TRIAE_CS42_6DL_TGACv1_528234_AA1712700 | 6.1 | Dhn4 | **Ta.Dhn4** |
| 23 | TRIAE_CS42_5BL_TGACv1_404666_AA1307790 | 1.9 | Dhn9 | **Ta.Dhn9** |
| 24 | TRIAE_CS42_5DL_TGACv1_432941_AA1395460 | 3.8 | Dhn9 | **Ta.Dhn9** |
| 25 | TRIAE_CS42_1BL_TGACv1_031362_AA0112350 | 4.7 | late embryogenesis abundant | **Ta.Wrab18** |
| 26 | TRIAE_CS42_1BL_TGACv1_031624_AA0117310 | 3.9 | late embryogenesis abundant | **Ta.LEA3** |
| 27 | TRIAE_CS42_1DL_TGACv1_062800_AA0220140 | 1.8 | late embryogenesis abundant | **Ta.LEA1** |
| 28 | TRIAE_CS42_7BL_TGACv1_579779_AA1909950 | inf | late embryogenesis abundant D-34-like | **Ta.LEA-D34-Like** |
| 29 | TRIAE_CS42_7AL_TGACv1_556273_AA1759750 | 5 | late embryogenesis abundant D-34-like | **Ta.LEA-D34-Lik** |
| 30 | TRIAE_CS42_3DS_TGACv1_273170_AA0929130 | 3.2 | Late embryogenesis abundant Lea14-A | **Ta.LEA14-A** |
| 31 | TRIAE_CS42_1DL_TGACv1_061688_AA0201730 | 1.3 | late embryogenesis abundant Lea14-A-like | **Ta.Lea14-A-like** |
| 32 | TRIAE_CS42_3AL_TGACv1_195540_AA0650890 | 1.4 | P5CS | **Ta.P5CS** |
| 33 | TRIAE_CS42_3B_TGACv1_225182_AA0805900 | 1.3 | P5CS | **Ta.P5CS** |
| 34 | TRIAE_CS42_3DL_TGACv1_250063_AA0861520 | 1.2 | P5CS | **Ta.P5CS** |
| 35 | TRIAE_CS42_1AL_TGACv1_003095_AA0047680 | -4.5 | proline dehydrogenase | **Ta.ProDH** |
| 36 | TRIAE_CS42_1BL_TGACv1_033285_AA0138440 | -3.8 | proline dehydrogenase | **Ta.ProDH** |
| 37 | TRIAE_CS42_1DL_TGACv1_061465_AA0196020 | -4.4 | proline dehydrogenase | **Ta.ProDH** |
| 39 | TRIAE_CS42_1AL_TGACv1_003095_AA0047680 | -4.5 | proline oxidase | **Ta.ProDH** |
| 40 | TRIAE_CS42_4BL_TGACv1_321295_AA1058570 | 2.4 | SOS2-like protein kinase PKS12 | **Ta.SOS2-like** |
| 43 | TRIAE_CS42_4DL_TGACv1_342497_AA1115230 | 1.4 | SOS2-like protein kinase PKS12 | **Ta.SOS2-like** |
| 44 | TRIAE_CS42_7DS_TGACv1_621622_AA2021390 | 1 | K^+^ transporter | **Ta.HAK25** |
| 45 | TRIAE_CS42_3AL_TGACv1_194519_AA0634830 | 1.14 | ABC transporter C | **Ta.ABAC15** |
| 46 | TRIAE_CS42_3B_TGACv1_224660_AA0799510 | 2.1 | Hexokinase | **Ta.HXK1** |
| 47 | TRIAE_CS42_4BL_TGACv1_320685_AA1046200 | 1.2 | Peroxidase | **Ta.POX** |
| 48 | TRIAE_CS42_1DL_TGACv1_061952_AA0206270 | 1 | Peroxidase | **Ta.POX** |
| 49 | TRIAE_CS42_1AL_TGACv1_006192_AA0055080 | 1.2 | Peroxidase | **Ta.POX** |
| 50 | TRIAE_CS42_1BL_TGACv1_034601_AA0145030 | 1.8 | Peroxidase | **Ta.POX** |
| 51 | TRIAE_CS42_1DL_TGACv1_061506_AA0197200 | 1.1 | Peroxidase | **Ta.POX** |
| 52 | TRIAE_CS42_1DL_TGACv1_063352_AA0226520 | 1 | Peroxidase | **Ta.POX** |
| 53 | TRIAE_CS42_2BL_TGACv1_132623_AA0438690 | 1.4 | Peroxidase | **Ta.POX** |
| 54 | TRIAE_CS42_2DL_TGACv1_157927_AA0502220 | 1.7 | Peroxidase | **Ta.POX** |
| 55 | TRIAE_CS42_2DL_TGACv1_157927_AA0502240 | 2.2 | Peroxidase | **Ta.POX** |
| 56 | TRIAE_CS42_5DS_TGACv1_456659_AA1475720 | inf | Glutaredoxin | **Ta.GRXC1** |
| 57 | Novel gene(TGACv1_scaffold_158087_2DL:10113-11146) | 3.4 | glutathione S-transferase | **Ta.GST** |
| 58 | Novel gene(TGACv1_scaffold_654146_U:49-427) | 3.4 | glutathione S-transferase | **Ta.GST** |
| 59 | TRIAE_CS42_6BS_TGACv1_513206_AA1634480 | 1 | Catalase | **Ta.CAT** |
| 60 | TRIAE_CS42_2AL_TGACv1_094377_AA0296730 | 1. 9 | non-selective cation channel | **Ta.GLR** |
